# Propolis as a functional food and promising agent for oral health and microbiota balance: A review study

**DOI:** 10.1002/fsn3.4216

**Published:** 2024-05-29

**Authors:** Arghavan Etebarian, Barbod Alhouei, Fatemeh Mohammadi‐Nasrabadi, Fatemeh Esfarjani

**Affiliations:** ^1^ Department of Oral and Maxillofacial Pathology, School of Dentistry Alborz University of Medical Sciences Karaj Iran; ^2^ Food and Nutrition Policy and Planning Research Department, National Nutrition and Food Technology Research Institute (NNFTRI), Faculty of Nutrition Sciences and Food Technology Shahid Beheshti University of Medical Sciences Tehran Iran

**Keywords:** functional food, honey, oral health, oral microbiota, propolis

## Abstract

Bee Propolis has been used for its therapeutic properties, including anti‐inflammatory, antibacterial, antifungal, and immune‐stimulating properties, for centuries as a functional food. This study reviewed the effectiveness of propolis as a functional food on oral‐related diseases as a rich bioflavonoid produced by honey bees. A literature search was conducted to identify studies published that investigated the effects of propolis on oral health and its ability to treat related diseases. The search was performed in electronic databases using relevant keywords. Initially, 3429 studies were identified through database searching, and based on the inclusion and exclusion criteria, 22 articles were eligible to be included. Reviewing the articles, propolis was recognized as a functional food and promising agent to balance oral microbiota and prevent oral diseases due to its effectiveness on related bacteria, its anti‐inflammatory properties, and its activity against *Porphyromonas gingivalis* and *Actinomyces Oris* allowed it to be an effective substance to prevent periodontal diseases. Based on our findings, Propolis is a desirable preventive option for various oral health conditions, including dental caries and periodontal diseases. Therefore, it is recommended to be consumed as a functional food in our daily diet, which can reduce the risk of oral disease and improve oral health.

## Graphical abstract

Propolis is a natural substance made by bees that has many health benefits, especially for oral diseases. This study reviewed 22 articles that showed how propolis can prevent and treat dental caries, periodontal diseases, and other oral problems by balancing the oral microbiota, fighting bacteria, and reducing inflammation.

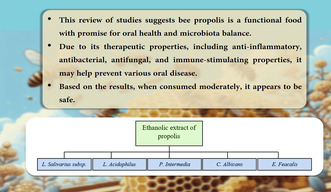

## 1 INTRODUCTION

Bee products are one of the beneficial substances; they are natural resources of antioxidants, including phenolic acids, flavonoids, terpenoids, etc. (Durazzo et al., 2021; Romário‐Silva et al., 2022). Among all bee products, propolis has been used for its therapeutic properties for centuries among ancient Egyptians, Persians, Greeks, Indians, and Australian Aborigines (Zulhendri, Lesmana, et al., 2022). Propolis, also called “bee glue,” is a resinous material with colors of dark brown to green and reddish brown that bees collect from twigs, buds, flowers, pollen, exudates, sprouts, and leaves and add their salivary secretions, which enriches it with *β*‐glucosidase (Alkhaled, 2022; Kang et al., 2022; Krishna et al., 2019; Zullkiflee et al., 2022). The name “propolis” has a Greek origin, “pro” means defend, and “polis” means city or community; now the function of this substance is vivid. Small gaps in the beehive are filled by propolis to protect against outside invaders like rain and bacteria. Also, it helps to maintain a balanced range of moisture and temperature (Kalia et al., 2022; Zullkiflee et al., 2022).

The chemical composition of propolis is strongly influenced by its geographical origin. For example, poplar bud exudates give European propolis its flavonoid aglycones and phenolic acids (Kasote et al., 2022). Red Cuban propolis derives its distinctive composition from *Clusia rosea* flowers (Bobiş, 2022), while green Brazilian propolis is rich in resins from *Baccharis dracunculifolia* leaves (Bobiş, 2022). Similarly, the different plant origins of propolis from Chile (Kasote et al., 2022), Venezuela (Kasote et al., 2022), Argentina, and Canada (*Eucalyptus*, *Ricinus*, *Clusia* spp., *Larrea nitida*) result in differences in chemical profiles such as those of phenylpropanes, epoxy lignans and polyisoprenylated benzophenones. The variety of propolis is essentially a reflection of the botanical environment in which it is collected (Kasote et al., 2022).

**FIGURE 1:**
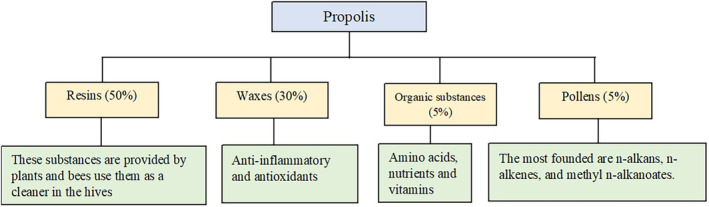
Major chemical components identified in propolis.

Propolis has nearly 500 bioactive compounds (Huang et al., 2014), which can be varied due to the different geographical regions: essential oils, pollens, amino acids, a wide variety of minerals including Iron, Zink, Copper, Calcium, vitamins (A, B1, B2, B6, C, D and E), flavonoids, aromatic substances and terpenes (Figure 1). Different concentrations are observed due to the botanical and geographical origin; for instance, propolis of Middle‐East origin showed the strongest antibacterial properties. Flavonoids, phenolic acids, and aromatic substances play crucial roles among the other components (Alkhaled, 2022; de Carvalho et al., 2019; Kalia et al., 2022; Kang et al., 2022; Krishna et al., 2019; Otręba et al., 2022). Propolis is a promising agent effective for dental caries (Deglovic et al., 2022; Ozan et al., 2007), bacterial fermentation, lactic acid production, and remaining biofilm, and, more significantly, *Streptococcus mutans* can start the process of dental caries (Kurek‐Górecka et al., 2022; Otręba et al., 2021; Yazdanian et al., 2022). Propolis has potent antibacterial properties that effectively combat a wide range of bacteria and fungi (Bouzahouane et al., 2021; Gebara et al., 2002). Its efficacy against periodontal culprits such as *Prevotella intermedia*, *Prevotella melaninogenica*, *Porphyromonas gingivalis*, *Actinobacillus actinomycetemcomitans*, *Capnocytophaga gingivalis*, and *Fusobacterium nucleatum* has been demonstrated in studies with minimum inhibitory concentrations (MICs) as low as 0.25 μg/mL (Gebara et al., 2002). Its effectiveness goes beyond dental health, inhibiting common infections such as *Candida albicans* and *Escherichia coli* (Gebara et al., 2002). Propolis contains a wide variety of bioactive substances such as flavonoids and phenolics that can disrupt microbial membranes and prevent their growth, which accounts for its broad‐spectrum action (De Vecchi & Drago, 2007). Periodontal disease is a chronic inflammatory multifactorial condition (Otręba et al., 2022; Sabbagh et al., 2023). The buildup of dental plaque is the main reason for these infections (Hajipour et al., 2022; Saeed et al., 2021). The most common form of gingival inflammation, plaque gingivitis, can be treated with proper dental hygiene and oral microbiota balance mouthwashes (Dehghani et al., 2019; Halboub et al., 2020; Krishna et al., 2019). Oral microbiota can maintain health conditions by balancing pH levels (by forming symbiotic biofilms) and affecting pathogens' growth. Also, they have benefits for other organs such as gut and cardiovascular homeostasis (Tuganbaev et al., 2022). Studies demonstrated that propolis contains compounds that could help maintain oral and gut microbiota balance (Alkhaled, 2022; Krishna et al., 2019).

**FIGURE 2:**
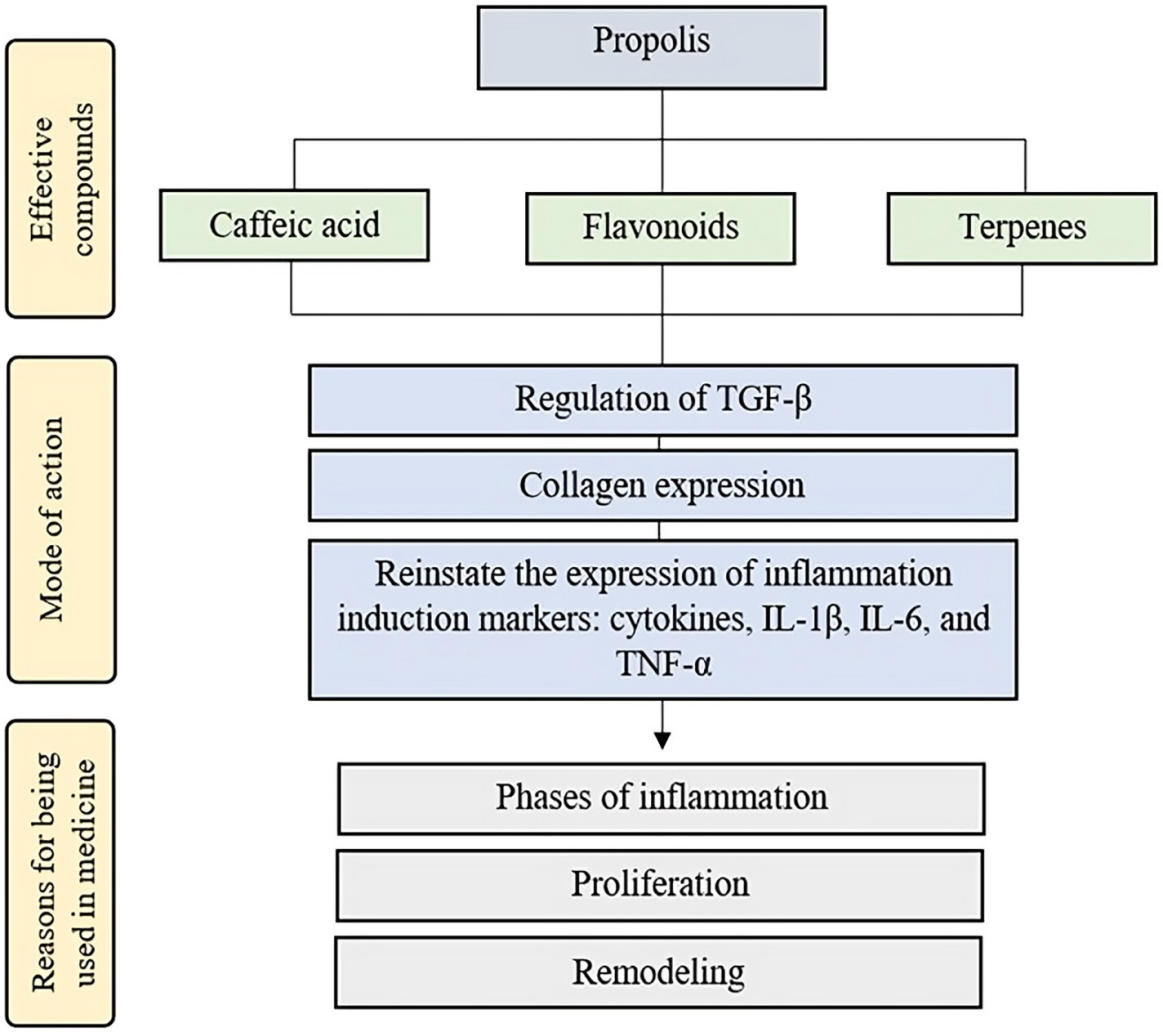
The possible pathways of propolis affecting inflammation.

Over the history of dental sciences, several kinds of drugs and oral care products have been created and used widely. The major drawback is the side effects, which limit their usage. This can be reinforced by the existence of multidrug‐resistant pathogens, which are getting stronger. Non‐chemical, plant‐derived medicines could address the problem. Thus, propolis has several dental health benefits due to its multiple antibacterial, anti‐inflammatory, and antioxidant properties (López‐Valverde et al., 2021) (Figure 2). Research suggests that it can reduce bacterial development and prevent tooth decay and gum irritation. This is especially true when used with Chlorhexidine (CHX) mouthwash, the antiseptic and disinfectant mouthwash. Propolis may also improve the treatment of gum disease by reducing the depth of the gum pocket and the oxidative stress that causes the disease (Lisbona‐González et al., 2021).

This is the first study reviewing the existing literature on propolis, a natural substance gaining popularity as a functional food. Its benefits on oral health were assessed by examining safety, comparing it to a mouthwash, and assessing its impact on periodontal disease and dental caries. Its potential to promote a healthy balance of mouth bacteria and its uses in food with its antioxidant and antibacterial properties were also investigated.

## 2 MATERIALS AND METHODS

### 2.1 Literature search

A literature search was conducted to identify studies published from 2000 to 20 July 2023 that investigated the effects of propolis on oral health and its ability to treat related diseases. The search was performed using electronic databases, including PubMed, Scopus, Web of Science, Science Direct, and Google Scholar, using relevant keywords including “propolis,” “oral health,” “honey,” “honey bee products,” “dental caries,” “periodontal diseases”, “Periodontitis”, “periodontal infection”, “oral cavity”, “propolis safety”, “functional food”, “Chlorhexidine”, “oral microbiota”, and “oral microbiome” alone or combined by “OR” and/or “AND.” Moreover, some reference lists of identified studies, related projects, congresses abstracts, dissertations, and relevant reviews were searched as gray literature to find, more likely, all eligible studies. The search strategy was designed to retrieve all relevant articles, including randomized controlled trials, cohort studies, case–control studies, and cross‐sectional studies. The titles and abstracts of the retrieved articles were screened for relevance, and the full texts of potentially eligible articles were reviewed for inclusion. Two independent reviewers performed data extraction and quality assessment, and any discrepancies were resolved through discussion or consultation with a third reviewer (Abedi et al., 2020; Hajipour et al., 2022; Mohammadi‐Nasrabadi et al., 2023; Sabbagh et al., 2023; Saeed et al., 2021). The EndNote X7 software (Thomson Research Soft, Philadelphia, PA) was used to import all the relevant articles. Duplicate studies were deleted.

### 2.2 Inclusion and exclusion criteria

For this review, abstracts were initially screened to exclude irrelevant studies. Full‐text articles were then assessed for eligibility, including those available in full text, written in English, provided detailed information on the medical properties of propolis and its effects on oral health, relevant studies, duplicate studies, and those not meeting the inclusion criteria, the relationship between propolis as a functional food and oral diseases, including dental caries and periodontal disease, comparison of the effectiveness of propolis with CHX.

### 2.3 Data extraction

Two independent reviewers performed data extraction using a standardized data extraction form. The extracted data included general characteristics of the study (the first author, year of publication, study design) and the main outcomes of the studies. Any discrepancies between the two reviewers were resolved through discussion and consensus.

### 2.4 Study selection process

**FIGURE 3:**
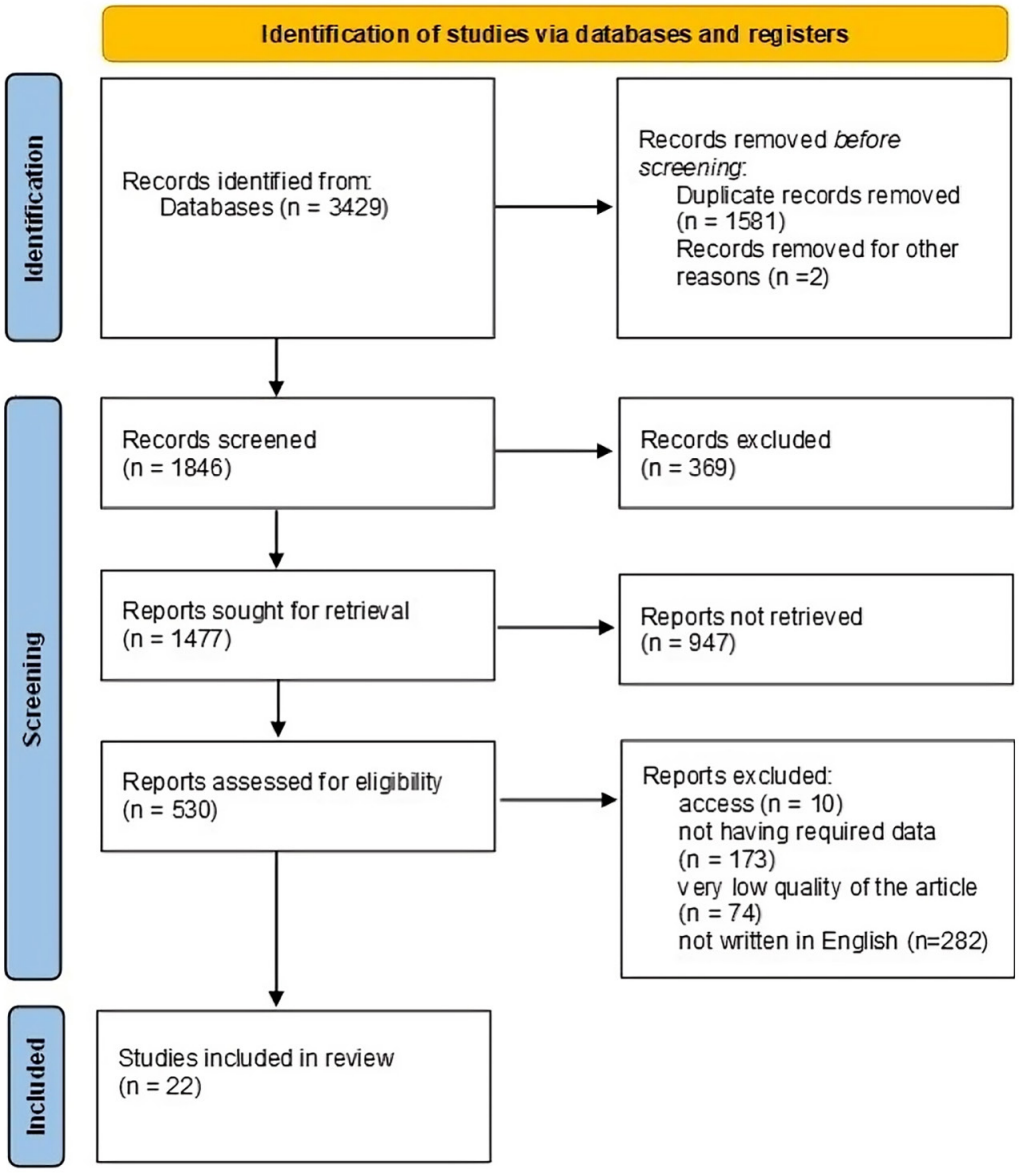
The flow chart of the literature search strategy and study selection process.

Initially, 3429 studies were identified through database searching. After removing 1583 duplicates, 1846 studies remained for screening by title and abstract. Of these, *n* = 1477 reports sought for retrieval; finally, 530 studies were assessed for eligibility, and 499 were excluded because of inaccessibility (*n* = 10), not having required data (*n* = 173), very low quality of the article (*n* = 74), not written in English (*n* = 282). Of the remaining publications in this review, 22 studies were deemed eligible (Figure 3).

### 2.5 Statistical analysis

The main strategy in the analysis was data synthesis. The heterogeneity of the included studies in terms of the study methods and outcome measurements hampered the possibility of a meta‐analysis. Therefore, the results were presented as qualitative and quantitative syntheses according to the type of the study.

## 3 RESULTS AND DISCUSSION

The results of the 22 studies showed that four studies (Dehghani et al., 2019; Krishna et al., 2019; Kumar et al., 2015; Kurek‐Górecka et al., 2022) showed a statistical reduction in the Plaque Index (PI) and gingival index (GI), one study (Alkhaled, 2022) reported a 75% reduction in oral microbiota count, two studies (Wiatrak et al., 2017, 2021) said the statistical decline in Approximal Plaque Index (API), two studies (Wiatrak et al., 2017, 2021) reported a statistical reduction in Simplified Oral Hygiene Index (OHI‐s), one study (Gebara et al., 2003) showed a promising reduction in the counts of anaerobic bacteria, three studies (Gebara et al., 2003; Kumar et al., 2015; Sanghani et al., 2014) showed a reduction in bacteria associated with periodontal diseases such as *Porphyromonas gingivalis*, one in *Prevotella intermedi* (Sanghani et al., 2014), *Tannerella forsythensis* and *Treponema denticola* (Kumar et al., 2015) and one study (López‐Valverde et al., 2021) represented reduction in Probing Pocket Depth (PPD) in Table 1.

**TABLE 1:** The summary of studies of propolis on oral health.

No.	Author, date	Study design	Extraction of propolis	Main outcomes
1	Sardana D, et al., 2013	Review study	N/R	Dental literature on propolis was based on in vitro studies or animal studies. Propolis may find a definitive role in one or more applications in dentistry
2	Mendonça IC, et al., 2013	Review study	N/R	The result of the literature review showed that propolis can cause allergic reactions in high consumption with a history of previous allergies
3	Hwu Y‐J & Lin F.‐Y, 2014	Meta‐analysis	Brazilian propolis/Hydro‐alcoholic solution of propolis (6%), 20% ethanol propolis extract	Significant reduction of *Candida*, decreasing the colonies and decreasing the number of *Streptococcus mutans*, fully treated edema, and erythema, Decreasing the frequency of outbreaks of stomatitis, improvement of QOL
4	Abbasi AJ, et al., 2018	Review study	Ethanolic extract of propolis	Propolis is useful for multiple purposes in oral health
5	de Carvalho C, et al., 2019	Review study	Brazilian propolis	Promises the usage of propolis against oral‐related microorganisms. Both green and red Brazilian propolis are effective for multifactorial oral cavities
6	Halboub E, et al., 2020	Systematic review	Water extract propolis and ethanol extract propolis	Reviewed and acknowledged that propolis has beneficial efficacy on periodontal diseases. And in some cases superior to CHX
7	López‐Valverde N, et al., 2021	Systematic Review with Meta‐Analysis	N\R	Probing pocket depth
				Difference (CI 95%): −0.67 (−0.84, −0.50)/Propolis is safe and can prevent periodontal disease, reduce probing pocket depth
8	Zulhendri F, Perera CO, et al., 2022	Systematic review	Australia, Brazil, Cameroon, Ghana, Indonesia, United Kingdom, China, Indonesia, Iran, Italy, Korea, Malaysia, Morocco, Taiwan, Turkey, Egypt, Methanolic Extract, Hydroethanolic Extract, Propolis extracted with gum Arabic, Ethanol Extract, Adjuvant in Experimental vaccine, Adjuvant of aqueous and Alcoholic extract, Propolis powder	Summarized the anti‐inflammatory effects of propolis by downregulation and inhibiting inflammatory factors and their associated proinflammatory cytokines
9	Zullkiflee N, et al., 2022	Review study	N\R	The results of this review showed propolis's constituents, biologically active compounds, and its efficacy in the treatment of human chronic diseases
10	Kalia A, et al., 2022	Review study	Cypress, Rosewood, Fabaceae, Brazil, European propolis, Russian, and Cuban propolis/Ethanol as solvent	Focused on the nutritional composition of propolis and the therapeutic uses of the substance in different health conditions and complications
11	Otręba M, et al., 2022	Systematic review	Brazilian propolis, Iran. Russia, Turkey, Korea, India, Czech, Germany, Irish, Peru, Cameroon, México, Australia, Poland, Bulgaria/Ethanolic extract, ethanolic extract (10%), crude ethanolic extract, ethanolic and aqueous extracts, alcoholic and hydroelectric extract, 33.3% ethanol propolis extract, 0.12% chlorhexidine digluconate, 80% ethanol, Milli‐Q water, ethyl acetate, acetone, water, DMSO extract of propolis, benzene, diethyl ether, and methyl chloride by the agar dilution method	The result of the review indicated that propolis demonstrates antimicrobial activity against the strains of bacteria that cause, for example, periodontitis, gingivitis, caries, subgingival plaque, and supragingival plaque
12	Kurek‐Górecka A, et al., 2022	Review study	Brazil/The ethanolic extract of propolis (EEP)	44.7% reduction in PI/Propolis is a useful substance that prevents the accumulation of dental plaque
13	Fraire‐Reyes IA, et al., 2022	Systematic review	N\R	Identified that propolis is a promising agent for treating periodontitis
14	Chatzopoulos GS, et al., 2022	Systematic review	N\R	Assessed the clinical effectiveness of propolis as an herbal
				Dental product on periodontitis. Moreover, herbal products may have comparable clinical outcomes with CHX with no relevant adverse effects
15	Ozan F, et al., 2007	In vitro	Turkey Yomra, Trabzon/10% w/v propolis, 25% v/v of 70% ethanol, 10% v/v propylene glycol and deionized water; (Romário‐Silva et al., 2022) 5% w/v propolis, 25% v/v of 70% ethanol, 10% v/v propylene glycol and deionized water; (Zulhendri, Lesmana, et al., 2022) 2.5% w/v propolis, 25% v/v of 70% ethanol, 10% v/v propylene glycol and deionized water; (Alkhaled, 2022) 1% w/v of 70% ethanol	Prepared a comparison between mouth rinses containing propolis and CHX. Demonstrated that CHX was more effective but, a proper concentration of propolis can be used as a mouth rinse to maintain oral health
16	Brailo V, et al., 2012	Case report	Propolis spray	A reported case of an 18‐year‐old female patient with allergic responses caused by propolis.
17	Krishna K, et al., 2019	Clinical Trial	5% propolis mouthwash	After 6 weeks (reduction percentage, *p* < .05)
				PI: 24.62%
				GI: 23.66%
				Propolis mouthwash exhibited a significant improvement
18	Dehghani M, et al., 2019	Clinical trial	30 g of propolis was combined with 100 mL of distilled water and then mixed, the mixing solution of propolis was 1% with a salt concentration of 0.25% with the essential oil of saffron and flavor	Mean Difference: (reduction)
				PI: −0.6, GI: −0.4/Propolis‐containing mouthwashes could be used as an alternative
19	Durazzo A, et al., 2021	Cross‐sectional	N/A	Clarified the antioxidant effects of propolis
20	Alkhaled A, 2022	In vivo	Syria/Ready‐to‐use 5% propolis solution produced by Tact Company for Essential Oils	74.78% reduction in oral microbiota count/Effectiveness of propolis mouth rinsing solution on oral flora
21	Kang W, et al., 2022	In vitro	Hawaii/50 mg of EEP was dissolved in 1 mL of 70% ethanol	Antimicrobial properties of propolis against oral pathogens to improve dental health by propolis
22	Yazdanian M, et al., 2022	In vitro	Iran/The ethanolic extract of propolis (EEP)	Determined the antifungal, antibacterial, and cytotoxicity characteristics of the propolis extracts from different areas of Iran

Abbreviations: API, approximal plaque index; CI, confidence interval; GI, gingival index; N/R, not reported; OHI‐s, simplified oral health index; PI, plaque index.

### 3.1 Functional food ingredients

The various medical properties of propolis have been of interest in the last two decades (Zulhendri, Perera, et al., 2022). The results of studies have categorized propolis into two different groups: one from tropical areas and another from temperate regions (poplar type) (Irigoiti, Navarro, et al., 2021; Vera et al., 2011). Although their main composition had the same proportions of resins, waxes, organic substances, and pollens (Anjum et al., 2019; Krishna et al., 2019), poplar‐type propolis mainly consisted of flavones, aromatic acids, and their esters (El‐Guendouz et al., 2019) and propolis from tropical areas has phenolics compounds and terpenes (Irigoiti, Navarro, et al., 2021). Propolis's tremendous properties have provided the opportunity to use it as a functional food ingredient, as follows:

- Antioxidant activity: Flavonoids and phenolic compounds are propolis's chief components; their antioxidant properties mainly rely on those substances (Irigoiti, Navarro, et al., 2021; Kalia et al., 2022). These compounds have a high antioxidant activity because they can give hydrogen atoms and electrons from an aromatic hydroxyl group to a free radical. The potential for charge delocalization within the aromatic ring double‐bond system also contributes to their antioxidant activity (Irigoiti, Navarro, et al., 2021).
- Antibacterial property: Propolis's activity against pathogens allowed it to be used in foodstuffs to reduce the bacterial load (Pobiega et al., 2019). There are several ways to elaborate on its antibacterial mechanism. Still, three of them were repeated mostly in studies: The change in membrane permeability, the prevention of protein synthesis, and the inhibition of bacterial movement (Santos et al., 2018; Vasilaki et al., 2019; Yazdanian et al., 2022). Also, it was reported by various studies that propolis had a synergistic activity with different antimicrobials (Dantas Silva et al., 2017), like amoxicillin (due to the presence of quercetin) (Almuhayawi, 2020) and chloramphenicol (Al‐Ani et al., 2018).
- Other pharmaceutical properties: Numerous studies acknowledged the effectiveness of propolis on different diseases, such as cardiovascular diseases (CVD) by benefiting the lipid profile (El‐Sharkawy et al., 2016; Samadi et al., 2017) and chronic kidney disease (CKD) by improving kidney function (Silveira et al., 2020). Nonalcoholic fatty liver disease (NAFLD) by reducing liver stiffness and high sensitivity C‐reactive protein (CRP) level (Soleimani et al., 2021; Zulhendri, Perera, et al., 2022), and diabetes by improving HbA1c, fasting blood sugar (FBS), fasting insulin level (Farida et al., 2023; Zakerkish et al., 2019).

### 3.2 Propolis used in the food industry

Propolis has been incorporated into food ingredients (majority techniques):

Spray drying (Busch et al., 2017; Šturm et al., 2019), Freeze drying (Šturm et al., 2019), Encapsulation (Jansen‐Alves et al., 2019; Keskin et al., 2019; Petrov et al., 2017), Chitosan (Barrera et al., 2015; Ebadi et al., 2019; Ezazi et al., 2021; Jonaidi Jafari et al., 2018; Piedrahíta Márquez et al., 2019) and Gelatin based coating (Marcinkowska‐Lesiak et al., 2021; Nessianpour et al., 2019; Ucak et al., 2020), Emulsification‐diffusion method (Correa et al., 2019; Mohammadi et al., 2019), and Co‐crystallization with sucrose (Irigoiti, Yamul, & Navarro, 2021), rice protein coating (Pires et al., 2019), polylactic acid films (Mascheroni et al., 2010), corn starch films (Ardjoum et al., 2023).

### 3.3 Dental caries and oral microbiota balance

A study found that propolis reduced the viability of *Streptococcus mutans*, inhibited *glucosyltransferase* activity, and slowed the progression of cavities in rats (Hayacibara et al., 2005). Further investigation revealed that apigenin, a component of propolis, was effective in preventing *glucosyltransferase* activity, while *t‐farnesol* was a potent antibacterial agent (Abbasi et al., 2018; Almuhayawi, 2020). Propolis has shown an antibacterial effect on *Streptococcus sobrinus*, *Streptococcus mutans* (*S. sorbins* and *S. mutans* are susceptible to pinocembrin, a flavonoid that exists in propolis (Almuhayawi, 2020)), and *Streptococcus cricetus*, the main pathogenesis factors contributing to dental caries (Alkhaled, 2022). Alkhaled (Alkhaled, 2022) showed a significant decrease in oral flora after using propolis, which has stronger antibacterial effectiveness than honey. Wiatrak et al. (2021) observed a substantial reduction in oral microbiota in participants using propolis‐containing toothpaste and a positive change in the quality of oral microbiota; moreover, consuming propolis increased the level of *Prevotella melaninogenica*, and *Campylobacter gracilis* which are dominant bacteria in healthy microbiome (Wiatrak et al., 2021; Wolff et al., 2019); in addition, a decline was observed in *Lactobacillus acidophilus* which is known as a responsible agent for dental caries (Wiatrak et al., 2017, 2021); concluding from the studies above, propolis by reducing pathogenic bacteria and increasing beneficial ones benefits the oral microbiota balance. Phenolic compounds are key in inhibiting *glycosyl‐transferase*, allowing bacteria like *Streptococcus mutans* to stick to the tooth surface. Flavonoid components (cinnamic acid (Almuhayawi, 2020)) of propolis bind to the *DNA gyrase* of bacteria like *Escherichia coli* to delay bacterial activity (Yazdanian et al., 2022).

### 3.4 Periodontal disease

Dental plaque can be distinguished as the main reason for periodontal diseases. Mainly, the biofilms of gram‐positive and gram‐negative bacteria with their metabolites cause a substance with a strong bond with teeth called “dental plaque” (Etebarian et al., 2023). To be more specific, dental plaque is a complex aggregation of oral bacteria, fungi, and other factors, and it has been proven that more than 10^11^ organisms of about 30 species are available per 1 mg of dental plaque (Dehghani et al., 2019; Hwu & Lin, 2014). The formation of dental plaque, irrespective of age, mainly depends on unsuitable oral hygiene and inappropriate diet (Halboub et al., 2020). There are several ways to maintain oral health, such as topical fluoride application, diet control, and probiotics intake such as mouthwashes (Alkhaled, 2022; Sardana et al., 2013). Propolis, by downregulating and inhibiting the inflammatory factors and their pro‐inflammatory cytokines, can act as a barrier against inflammation caused by infectious diseases (Zulhendri, Lesmana, et al., 2022). 50 μg/mL Hawaiian ethanolic extract of propolis (EEP) showed antibacterial effects against *Actinomyces oris* and *Porphyromonas gingivalis* (*P. gingivalis* is susceptible to quercetin, a flavonoid in propolis (Almuhayawi, 2020)), Explaining propolis's effectiveness against both Gram‐positive and Gram‐negative bacteria. In addition, *Porphyromonas gingivalis* (76) and *Actinomyces oris* are widely present in dental plaque, and the antibacterial effect of propolis against these species (minimum inhibitory concentration (MIC) μg/mL for *Porphyromonas gingivalis* was 8.0 and for *A. oris* was 10.6 (Kang et al., 2022)) this tends to be a reason for using propolis as an oral care product to prevent periodontitis (Kang et al., 2022).

Some studies mentioned a greener way to extract propolis active compounds, which is NADES (natural deep eutectic solvents) rather than ethanolic and water‐based solvents, which are the most often used techniques (Yurt, 2023). Tzani et al. (2022) explored NADES for Greek propolis, achieving extracts rich in phenolics and antioxidants. Their winning NADES formula combined choline chloride and glycerol. Chaves et al. (Funari et al., 2019) compared NADES to traditional solvents (ethanol, water) for green propolis extraction. NADES extracts shone with impressive phenolic content and antioxidant capacity. Tsiaka et al. (Vasileva et al., 2022) further solidified NADES' potential by effectively extracting bioactive compounds from propolis alongside other natural sources like *Sideritis scardica* and *Plantago major*. Tzani et al. (Alpat et al., 2023) delved deeper, optimizing propolis extraction with NADES. They identified a 24:1 solvent‐to‐propolis ratio and 75.71% NADES composition as the ideal combination.

### 3.5 The comparison between the use of propolis and chlorhexidine in oral health care

**FIGURE 4:**
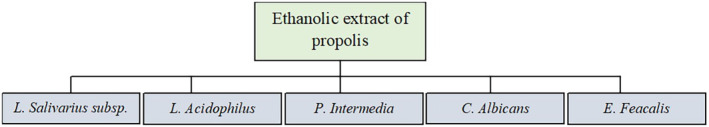
The ethanolic extract of propolis (EEP) against susceptible bacteria.

**FIGURE 5:**
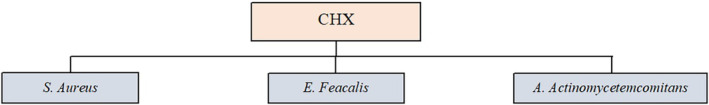
Chlorhexidine (CHX) against susceptible bacteria (Mendonça et al., 2013).

Chlorhexidine (CHX) containing mouthwashes are widely used in periodontal diseases due to their approved prompt. However, the cytotoxicity and side effects, including altered taste sensations, teeth staining, soreness, and dryness, are still under debate (Alkhaled, 2022; Chatzopoulos et al., 2022). Although there are many chemically effective oral care products, their side effects conceal their positive criteria; therefore, using non‐chemical alternatives is now of great interest (Chatzopoulos et al., 2022). There were contradictory pieces of evidence on the comparison of propolis and CHX efficacy; several studies acknowledged that propolis has equal or even superior effects than CHX (Halboub et al., 2020). One study examined the effectiveness of propolis compared to CHX, and its bactericidal effect was stronger than CHX on *Prevotella intermedia* (Akca et al., 2016). The summary of different bacteria that are affected by propolis and CHX is available in Figures 4 and 5. A study in 2019 concluded that chronic gingivitis could be treated with propolis 5% mouthwash with improved significant results (Krishna et al., 2019). In addition, a study concluded that papillary bleeding can be better controlled by consuming propolis mouthwash compared to CHX (López‐Valverde et al., 2021). An analysis performed in 2007 showed that a mouthwash containing propolis with concentrations of 5%, 2.5%, and 1% exhibited no cytotoxicity and 10% with mild cytotoxicity, compared to CHX 0.2% which had moderate cytotoxicity (Ozan et al., 2007).

### 3.6 Propolis safety

WHO describes adverse drug reaction as any unintended or adverse side effect resulting from a medicine taken at a dose normally used in humans for disease prevention, diagnosis, treatment, prophylaxis, or change of physiological functioning (Mendonça et al., 2013). All synthetic or natural drugs might produce adverse effects, and propolis is not an exception. The safety of propolis is distinguished by potential toxicity and allergic reactions. It contains components that have the potential to be toxic, like caffeic acid (Abbasi et al., 2018), benzyl benzoate (may cause CNS disorders), benzoic acid (when it reacts with vitamin C, produces carcinogenic benzene), and phenol (long‐term in high concentration may harm the heart, kidneys, and liver and lungs), but previous studies proved that propolis in a safe dosage (70 or 1.4 mg/kg of body weight) does not have toxicity (Abbasi et al., 2018; Mendonça et al., 2013). Many allergic reactions were reported, including dermatological (Walgrave et al., 2005) or respiratory symptoms, digestive tract complications (Menniti‐Ippolito et al., 2008), Cheilitis and posterior dermatitis (Brailo et al., 2012), stomatitis, perioral eczema, and dyspnea. The most common allergic reaction is contact dermatitis, which is limited to the applied area (Mendonça et al., 2013).

## 4 FUTURE RESEARCH

This is the first comprehensive review done to determine propolis's effectiveness as a functional food in oral microbiota balance and promoting oral health, assessing its safety, and comparing it to CHX. However, there was a lack of longitudinal studies that provided a better understanding of this association. Future studies on the appropriate amount and type of propolis are recommended. Further intervention with standardized protocols and long‐term follow‐up will be needed to determine its optimal dosage, time of application, method of preparation, and effects on allergic reaction.

## 5 CONCLUSION

The results highlighted that propolis is a desirable agent that can be used to promote oral health conditions, including dental caries, due to its effectiveness on *Streptococcus sobrinus*, *Streptococcus mutans*, *Streptococcus cricetus*, and *Escherichia coli*. It may have a beneficial role in prevention of the periodontal disease by affecting *Porphyromonas gingivalis* and *Actinomyces oris*. Moreover, it can enrich healthy oral microbiota with minimal side effects compared to chlorohexidine. Therefore, it is recommended to be consumed as a functional food in our daily diet to reduce the risk of oral disease and improve oral health.

## AUTHOR CONTRIBUTIONS

**Arghavan Etebarian:** Conceptualization (equal); data curation (equal); formal analysis (equal); funding acquisition (equal); investigation (equal); methodology (equal); project administration (equal); resources (equal); software (equal); supervision (equal); validation (equal); visualization (equal); writing – original draft (equal); writing – review and editing (equal). **Barbod Alhouei:** Conceptualization (equal); data curation (equal); formal analysis (equal); funding acquisition (equal); investigation (equal); methodology (equal); project administration (equal); resources (equal); software (equal); supervision (equal); validation (equal); visualization (equal); writing – original draft (equal); writing – review and editing (equal). **Fatemeh Mohammadi‐Nasrabadi:** Conceptualization (equal); data curation (equal); formal analysis (equal); funding acquisition (equal); investigation (equal); methodology (equal); project administration (equal); resources (equal); software (equal); supervision (equal); validation (equal); visualization (equal); writing – original draft (equal); writing – review and editing (equal). **Fatemeh Esfarjani:** Conceptualization (equal); data curation (equal); formal analysis (equal); funding acquisition (equal); investigation (equal); methodology (equal); project administration (equal); resources (equal); software (equal); supervision (equal); validation (equal); visualization (equal); writing – original draft (equal); writing – review and editing (equal).

## FUNDING INFORMATION

This research received no external funding.

## CONFLICT OF INTEREST STATEMENT

All authors work/study in academic educational and research centers in Iran and declared no conflict of interest.

## ETHICAL APPROVAL

Not applicable.

## Data Availability

The datasets analyzed during the current study are available from the corresponding author upon reasonable request.
